# Uncovering the Role of *N*-Glycan Occupancy on the Cooperative Assembly of Spike and Angiotensin Converting Enzyme 2 Complexes: Insights from Glycoengineering and Native Mass Spectrometry

**DOI:** 10.1021/jacs.3c00291

**Published:** 2023-03-31

**Authors:** Tarick J. El-Baba, Corinne A. Lutomski, Sean A. Burnap, Jani R. Bolla, Lindsay A. Baker, Andrew J. Baldwin, Weston B. Struwe, Carol V. Robinson

**Affiliations:** † Physical and Theoretical Chemistry Laboratory, Department of Chemistry, University of Oxford, South Parks Road, Oxford OX1 3TA, U.K.; ‡ The Kavli Institute for Nanoscience Discovery, Dorothy Crowfoot Hodgkin Building, South Parks Road, Oxford OX1 3QU, U.K.; § Department of Biochemistry, University of Oxford, Oxford, OX1 3QU, U.K.

## Abstract

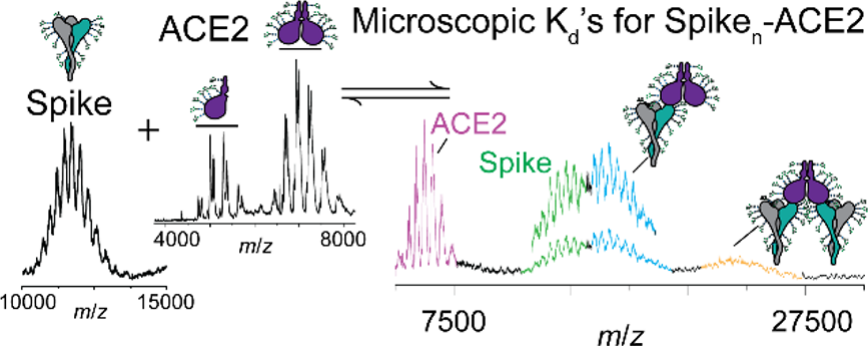

Interactions between the SARS-CoV-2 Spike protein and ACE2 are one of the most scrutinized reactions of our time. Yet, questions remain as to the impact of glycans on mediating ACE2 dimerization and downstream interactions with Spike. Here, we address these unanswered questions by combining a glycoengineering strategy with high-resolution native mass spectrometry (MS) to investigate the impact of *N*-glycan occupancy on the assembly of multiple Spike-ACE2 complexes. We confirmed that intact Spike trimers have all 66 N-linked sites occupied. For monomeric ACE2, all seven N-linked glycan sites are occupied to various degrees; six sites have >90% occupancy, while the seventh site (Asn690) is only partially occupied (∼30%). By resolving the glycoforms on ACE2, we deciphered the influence of each *N*-glycan on ACE2 dimerization. Unexpectedly, we found that Asn432 plays a role in mediating dimerization, a result confirmed by site-directed mutagenesis. We also found that glycosylated dimeric ACE2 and Spike trimers form complexes with multiple stoichiometries (Spike-ACE2 and Spike_2_-ACE2) with dissociation constants (*K*_d_s) of ∼500 and <100 nM, respectively. Comparing these values indicates that positive cooperativity may drive ACE2 dimers to complex with multiple Spike trimers. Overall, our results show that occupancy has a key regulatory role in mediating interactions between ACE2 dimers and Spike trimers. More generally, since soluble ACE2 (sACE2) retains an intact SARS-CoV-2 interaction site, the importance of glycosylation in ACE2 dimerization and the propensity for Spike and ACE2 to assemble into higher oligomers are molecular details important for developing strategies for neutralizing the virus.

## Introduction

The surface of SARS-CoV-2 is decorated with class I fusion Spike glycoproteins, which are central mediators of viral infection. Spike itself is a ∼150 kDa membrane protein that forms trimers on the surface of a virion. Each virion contains ∼20–40 trimers.^1^ The Spike amino acid sequence can be divided into two domains, S1 and S2, which are integral to cell attachment and membrane fusion, respectively. S1 of each protomer contains an N-terminal domain (NTD) and a receptor binding domain (RBD), the latter of which stochastically adopts an “up” conformation, which facilitates binding to the host’s angiotensin converting enzyme-2 (ACE2) receptor, thereby initiating the cell entry process.^2−4^ Along with serving as the SARS-CoV-2 receptor, ACE2 plays key roles in regulating the renin-angiotensin system (RAS).^5^ Cellular ACE2 (cACE2), which is the main target of SARS-CoV-2, is cleaved from the cell surface by the metalloprotease ADAM17, which leads to the release of an enzymatically active soluble form of ACE2 (sACE2) into the plasma.^6^ The SARS-CoV-2 interaction site is maintained in sACE2, and indeed, sACE2 is a powerful SARS-CoV-2 neutralizing agent.^7^ However, the impact of circulating sACE2 in COVID19 severity and treatment is controversial.^8−10^ Recent reports state that concentrations of sACE2 may be influenced by regulatory pathways that are related to the severity of COVID-19.^11^ In addition, the potential therapeutic use of sACE2 (and engineered sACE2 analogues) via neutralization of Spike RBDs on the surface of circulating SARS-CoV-2 has been clearly demonstrated.^8,12,13^ Therefore, understanding the molecular details of Spike and ACE2 interactions is both topical and important for understanding potential therapeutic roles of sACE2.

Atomic structures of Spike engaged with ACE2 provide a structural basis for the earliest steps in the viral recognition process.^3,4,14−16^ Furthermore, biophysical studies such as hydrogen–deuterium exchange mass spectrometry have generated descriptive views of the dynamics involved in the interactions between Spike and sACE2.^17^ Atomistic molecular dynamics (MD) simulations have implicated specific *N*-glycan sites in mediating the up/down sampling of Spike RBDs,^18^ and a recent course-grained study found that Spike and ACE2 form complexes with multiple stoichiometries across parallel lipid bilayers.^19^ These MD simulations, however, are difficult to validate experimentally. Furthermore, it is challenging to resolve heterogeneous glycans and consequently to probe their structure-specific involvement in glycoprotein interactions without abrogating an N-linked glycosylation site entirely. Several studies have suggested that N-linked glycans (on the virus or host) play important roles in SARS-CoV-2 infectivity,^20−24^ including the recognition of the ACE2 receptor by Spike.^25^ In addition, results have demonstrated the importance glycans and glycoproteins in SARS-CoV-2.^26−30^ Yet, existing structural approaches have limitations in their ability to resolve directly the impact of individual glycans on Spike protein interactions. In addition to considering glycosylation of Spike, ACE2 exists in monomeric and dimeric forms and has seven *N*-glycosylation sites. Since *N*-glycan structures are heterogeneous, the impact of glycosylation on the ACE2 monomer–dimer equilibrium warrants further investigation since it may lead to some of the contradictory results that have been reported.^8−10,12^ Moreover, the impact of ACE2 glycosylation on the stoichiometry of complexes, and their binding affinity for glycoforms of Spike, is a multifactorial problem that requires new approaches for investigation.

Glycosylation profiles are typically determined by mass spectrometry (MS)-based glycoproteomic methodologies.^31,32^ In the context of SARS-CoV-2 Spike, the overall consensus is that the protein is heavily glycosylated.^33−38^ Resolving glycan-specific interactions, however, is exceedingly complex due to two factors: variability of glycan structures at a given site (microheterogeneity) and the presence or absence of glycans at a given site (macroheterogeneity). An attractive alternative to alleviate this heterogeneity is to use *glycoengineering*. While this term encompasses both deglycosylation (or ″glycan remodeling″) via enzymes^39−41^ and *in vivo* engineering by deleting genes/inhibiting enzymes,^42,43^ both cases result in the generation of a glycoprotein with a single glycosylation profile. To this end, genetic knock-out of *N*-acetyl-glucosaminyltransferase I (GNTI) in HEK cells to limit *N*-glycans to high-mannose type glycans with sequence Man_5_GlcNAc_2_ is a protocol routinely used in structural studies.^44,45^ The use of GNTI^–/–^ in conjunction with native MS, to evaluate glycoprotein interaction strengths, has not yet been reported.

High-resolution native MS is an emerging method for uncovering glycan-specific biomolecular interactions, beyond current ensemble-based structural methods.^46,47^ However, the expansive repertoire of possible glycoforms on a single N-linked site has limited its application to all but relatively small (<150 kDa) glycoproteins with limited heterogeneity.^39−43,48,49^ Given that the distribution of molecular masses of a given glycoprotein can differ by as little as 146 Da (i.e., a single fucose residue), in the context of an intact Spike trimer (mass of ca. 500 kDa), this will result in unresolved peaks in mass-to-charge ratio (*m*/*z*) spectra. Charge detection MS (CDMS)^50−52^ and mass photometry^53^ circumvent issues associated with measuring the molecular mass of large glycoprotein assemblies. These emerging technologies are however currently limited in their ability to resolve individual glycosylation states. Thus, it has not been possible to link directly the impact of specific glycoforms on binding, making it impossible to evaluate their effects on intact Spike-ACE2 interactions.

Here, we address this knowledge gap in glycan specificity in Spike-ACE2 interactions, focusing on the involvement of *N*-glycans in complex assemblies. To do this, we adapted a mammalian protein expression strategy to generate glycoengineered ACE2 (∼190 kDa) and Spike (>500 kDa), making them absent of *N*-glycan microheterogeneity and amenable to native MS. We find that all 66 N-linked sites on trimeric Spike are occupied. Similarly, we find that 12 of the 14 N-linked sites on dimeric ACE2 are >90% occupied. Glycoproteomic analysis of the glycoengineered ACE2 enabled us to localize the sites with partial glycan occupancy and prompted us to carry out site-directed mutagenesis to explore the impact of selected *N*-glycans in mediating ACE2 dimerization. We were also able to show that Spike and ACE2 complexes, lacking *N*-glycan microheterogeneity, (i.e., with all sites occupied by Man_5_GlcNAc_2_) are able to interact with nanomolar affinities. Unexpectedly, we found that the most favorable Spike-ACE2 complex is an assembly comprising two Spike trimers bridged by an ACE2 dimer. Taken together, our findings demonstrate that ACE2 glycoforms regulate dimerization. Furthermore, we find that sACE2 binds multiple Spike trimers with exceptionally high affinity, providing insights into the neutralizing abilities of circulating sACE2.

## Results and Discussion

### Deciphering Glycoforms on Trimeric Spike Proteins

**Figure 1:**
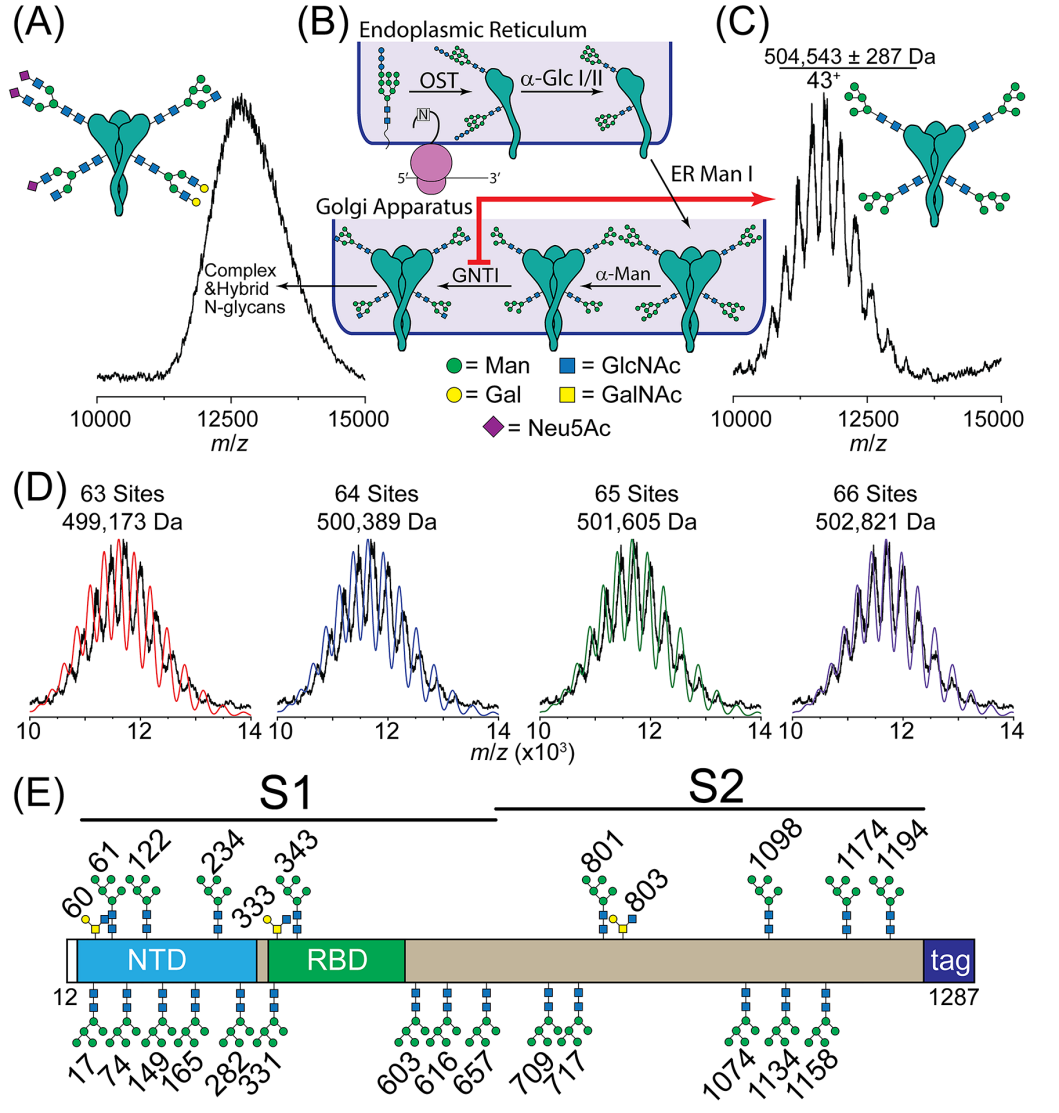
Native mass spectrometry of trimeric Spike. (A) An unresolved native mass spectrum of Spike is observed consistent with heterogeneous *N*-glycans. (B) Cartoon depiction of N-linked glycan processing in the ER and Golgi. After the preassembled GlcNAc_2_Man_9_Glc_3_ glycan is transferred to a nascent Spike protein, resident ER enzymes process the immature glycan. Once in the Golgi, the glycan structures are trimmed to Man_5_GlcNAc_2_, after which GNTI affixes a GlcNAc to Man_5_GlcNAc_2_, priming the glycan for further processing to complex and hybrid-type structures. (C) Well-resolved charge state envelope observed in the native mass spectrum of the Spike trimer expressed in GNTI^–/–^ HEK293 cells (red arrow in B). A mass of 504,543 ± 283 Da was measured. (D) Charge state envelopes, with varying extents of glycan occupancies, were simulated and compared with experimental spectra to evaluate *N*-glycan occupancy. Trimers harboring Man_5_GlcNAc_2_ at each of the 66 N-linked glycosylation sites are consistent with the measured spectrum and mass. (E) Glycoproteomic analysis validated the glycan occupancy of all 66 N-linked sites.

To explore molecular interactions between Spike proteins, we first recorded native mass spectra of the trimeric Spike protein harboring hybrid, complex, and oligomannose type N-linked glycans at each of the 66 possible sequons (Figure 1). We anticipated a molecular weight of ca. 520 kDa from our previous CDMS study.^50^ Charge states were not resolved in our spectrum due to the expansive glycoform heterogeneity that arises during transit through the secretory pathway in the cell, leading to many mass distributions (differing by as little as 157 Da). This generates overlapping mass-to-charge (*m*/*z*) ratios, precluding mass measurement. To overcome this problem, we expressed the Spike protein in HEK293 GNTI^–/–^ cells,^44,54^ thereby limiting N-linked glycan processing to produce a homogenous Spike with only Man_5_GlcNAc_2_ structures with a mass of 1216 Da (Figure 1B). We opted for this expression strategy over metabolic treatment (e.g., inhibition of ER mannosidases) or enzymatic glycan trimming, as genetic ablation ensures homogenous *N*-glycans on glycoproteins that retain full activity.^44,54^ As the genetic knockout does not perturb the co-translational addition of the preassembled *N*-glycan, occupancies determined by this strategy will be identical to those found in the parental HEK293 cells. After purification of the secreted trimeric ectodomains, a well-resolved charge state envelope was observed (Figure 1C). The charge states were assigned (see Supporting information) and enabled us to determine the mass, *m* = 504,543 ± 287 Da. The measured molecular weight is within 1% of 502,821 Da, the mass anticipated for trimers fully occupied by Man_5_GlcNAc_2_. The mass and average charge are within experimental error of our previous CDMS measurement.^50^

Next, we explored the possibility that fewer than 66 N-linked sites were occupied on Spike trimers^33−37^ by simulating native mass spectra for species with different N-linked occupancies. With 66 possible N-linked sites per trimer and only one allowable glycan structure (Man_5_GlcNAc_2_), there are 2^66^ possible glycoforms. Therefore, we limited the array of possibilities in our simulation by only considering glycoforms differing by ≤1.0% since the deviation between the measured and anticipated molecular mass was <1% (Figure 1D). To further simplify the spectral comparisons, a constant, empirically determined peak width was used to simulate the entire charge state envelope. This approximation provides a straightforward and visual means for spectral matching. Since broad peaks will dilate more readily at lower charge states (higher *m*/*z* values), better correspondence with the peak intensities at lower *m*/*z* values is expected. Considering first the simulations for 63, 64, and 65 N-linked sites, poor correspondence is observed with the experimental data. We propose that these species with 63–65 N-linked sites, if present, represent minor components of the overall distribution of Spike trimers. Agreement between the experimental data and the simulations improves with increasing occupancy of N-linked sites. Simulating occupancy at 66 N-linked sites, as identified by glycoproteomics (Figure 1E), corresponds well with the measured charge state distribution.

We corroborated our assignments using glycoproteomics to confirm the presence of *N*-glycans on peptides isolated from digested Spike proteins (Figure 1E). Consistent with our previous study,^55^ we found evidence for Man_5_GlcNAc_2_ glycans at all N-linked sites. We quantified the relative *N*-glycan occupancy at each site and found that only a minor fraction of peptides containing Asn657 were unoccupied (Figure S1). These *N*-glycan occupancies are consistent with previous glycomics and glycoproteomics studies.^33−38^ Thus, our findings identify that Spike trimers generated from GNTI^–/–^ preferentially assemble from protomers harboring full N-linked glycan occupancy.

### ACE2 Glycoforms Impact Dimerization

**Figure 2:**
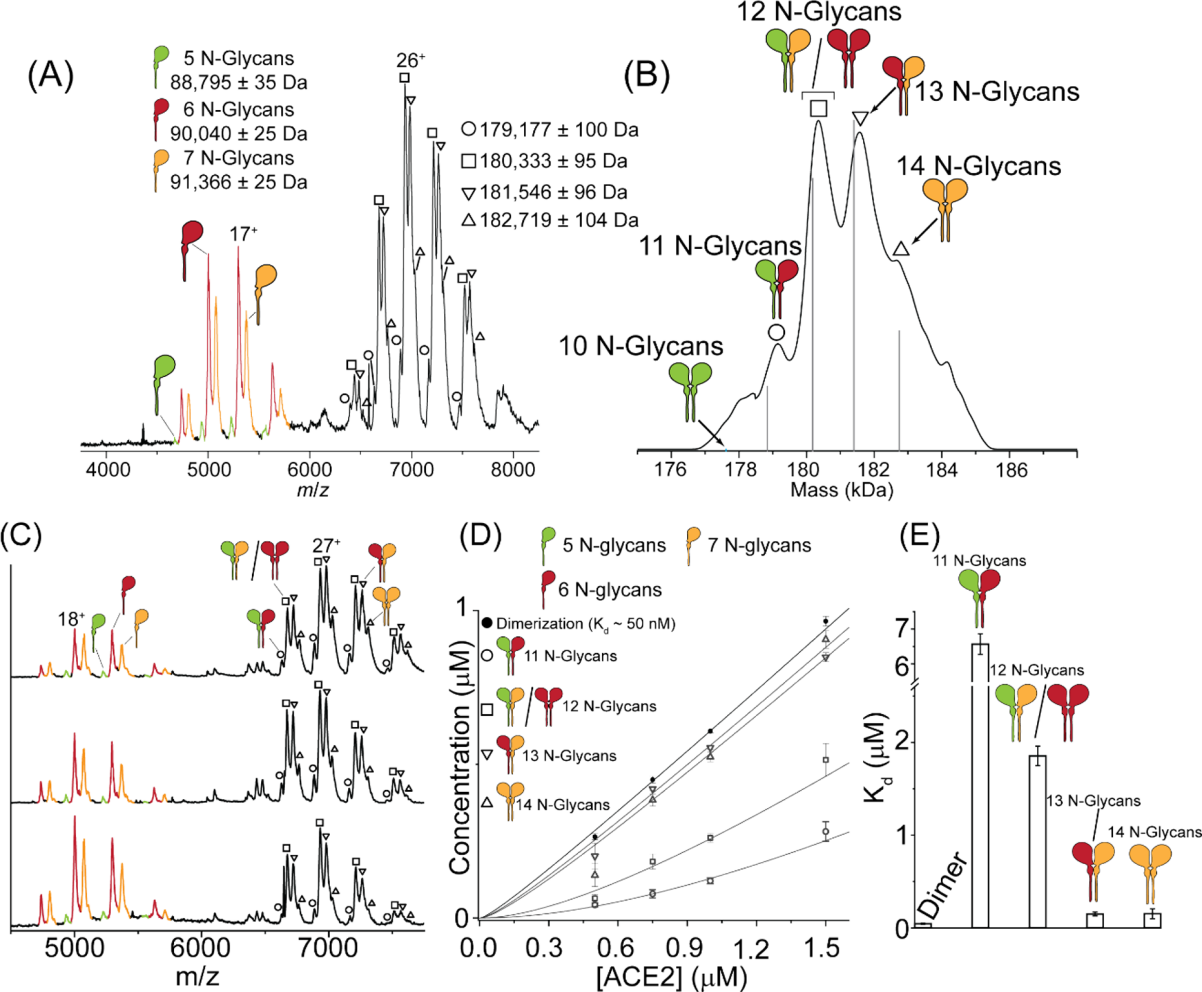
*N*-glycan occupancy mediates ACE2 dimerization. (A) Native mass spectrum of ACE2 identifies monomers and dimers with varying extents of N-linked glycan occupancy. (B) Zero charge state deconvoluted mass spectrum of ACE2 dimers. Five peaks with masses between 178 and 185 kDa are consistent with a simulated mass spectrum (gray lines) for ACE2 dimerization from specific glycoforms on the monomer. (C) Representative native mass spectra of ACE2 at 0.5, 1.0, and 1.5 μM (bottom to top). (D) Plot of the dimer concentration at each ACE2 protein concentration for the different dimer glycoforms. Solid lines show the results of fitting a monomer–dimer equilibrium model. (E) bar chart depicting microscopic *K*_d_ values for each dimeric ACE2: global dimerization (i.e., the *K*_d_ representing the overall monomer–dimer equilibrium): 45 ± 7 nM, 11 *N*-glycans (green-red): 6.56 ± 0.30 μM, 12 *N*-glycans (green-yellow and red-red): 1.86 ± 0.10 μM, 13 *N*-glycans: 150 ± 21 nM,14 *N*-glycans: 152 ± 53 nM). Error bars denote the standard deviation from *n* = 3 independent replicates.

We used the same expression strategy to generate soluble ACE2 (sACE2) ectodomains consisting of Man_5_GlcNAc_2_ N-glycoforms. After purification, we recorded native mass spectra of sACE2 (Figure 2A) and observed two well-separated charge state envelopes, one centered at 17^+^ and another at 26^+^. These two envelopes are assigned to monomers and dimers of sACE2 (ca. 90 and 180 kDa, respectively). Within these respective charge state envelopes, split peaks are observed with a mass that corresponds to different *N*-glycan occupancies (i.e., mass differences of 1216 Da assigned to a single Man_5_GlcNAc_2_).

To understand the distribution of ACE2 *N*-glycan occupancies, we focused on charge states assigned to monomeric ACE2 (centered at ∼*m*/*z* 5500, Figure 2A). We obtained deconvoluted molecular masses of 88,795, 90,040, and 91,366 Da. These masses are in close agreement to those anticipated for monomeric ACE2 harboring five, six, and seven N-linked glycans (Table S1). Turning to the charge state envelopes assigned to dimeric ACE2 (∼*m*/*z* 7500), we found at least four well-resolved charge state distributions corresponding to molecular mases of 179,117, 180,333, 181,546, and 182,719 Da. ACE2 has seven predicted N-linked sites, all of which have been found to harbor glycosylation by glycoproteomic analysis.^20^ Consistent with these studies, our glycoproteomic analysis of enzymatically digested ACE2 found evidence for high (>90%) occupancy at six of the seven N-linked sites (Figure S2). All sites contain occupancy >95%, except for Asn432 (∼90%) and Asn690 (∼30%). These four charge state distributions are therefore assigned to dimeric ACE2 species harboring 11–14 N-linked glycans, respectively.

The peaks for the ACE2 glycoforms are well resolved, which allowed us to explore the role that glycans have on dimerization. Specifically, we explored the hypothesis that the formation of dimeric ACE2 is sensitive to monomeric ACE2 glycosylation. Six possible organizations of dimers can arise from the three different glycoforms of monomeric ACE2 (with 5, 6, and 7 *N*-glycans). We first explored the possibility that the different dimeric forms arise from combinatorial association of the three different glycoforms on the monomer. We used a polynomial expansion to simulate a predicted pattern of dimeric ACE2 from the peak intensities of the monomeric ACE2 glycoforms. The results of the simulation show that the anticipated molecular weights and intensities for five dimeric ACE2 species are close to those determined experimentally (Figure 2B). The observation that the abundance of dimers with 10 N-linked glycans is only at trace levels is recapitulated by this polynomial expansion. Furthermore, the simulated abundances for dimers with 12 and 13 *N*-glycans, the most abundant glycoforms that can arise from the three ACE2 proteoforms, are similar to the abundances observed experimentally. However, there are some minor differences in the abundance of the 12 *N*-glycans ACE2 dimer, which is of higher intensity than that of the 13 *N*-glycans, whereas the simulated abundances are reversed. These observations lead us to consider the hypothesis that dimerization is not a stochastic event but could be impacted by the organization of *N*-glycans.

To explore this hypothesis, we estimated the concentration dependence of these events by calculating dissociation constants of each of the distinct glycosylated dimers (from 10–14 *N*-glycans). We recorded native mass spectra at four different ACE2 concentrations from 0.500–1.5 μM. At an ACE2 concertation of 0.500 μM the spectrum is populated predominantly by monomeric ACE2 (Figure 2C). As the concentration of protein is increased, monomer peaks decreased, concomitant with increased intensities of dimeric ACE2 glycoforms. We quantified the concentration of dimeric ACE2 across our serial dilution and fitted the resulting datasets to a monomer-dimer equilibrium binding model to determine the global dissociation constant (*K*_d_) dimerization, *K*_d_ = 45 ± 7 nM (Figure 2D). This indicates that even when *N*-glycan involvement is not considered, ACE2 has a high preference for dimerization.

We then extracted the individual abundances of each dimer glycoform at the different ACE2 concentrations to determine their *K*_d_ values. As we do not observe evidence for homodimerization between ACE2 monomers with five occupied N-linked sites, we surmise that this *K*_d_ is unfavorable. The formation of dimers with 11 N-linked glycans is relatively weak *K*_d_ = 6.56 ± 0.30 μM. By contrast, formation of dimers with 12 *N*-glycans is more favorable, *K*_d_ = 1.86 ± 0.10 μM. Yet more favorable are dimers of ACE2 with 13 and 14 *N*-glycans, with dramatic decreases in *K*_d_ (0.150 ± 0.021 and 0.152 ± 0.053 μM, respectively). These *K*_d_ values support our hypothesis that glycan occupancy is mediating ACE2 dimerization (Figure 2E).

### Differential Glycosylation Influences Spike-ACE2 Assemblies

**Figure 3:**
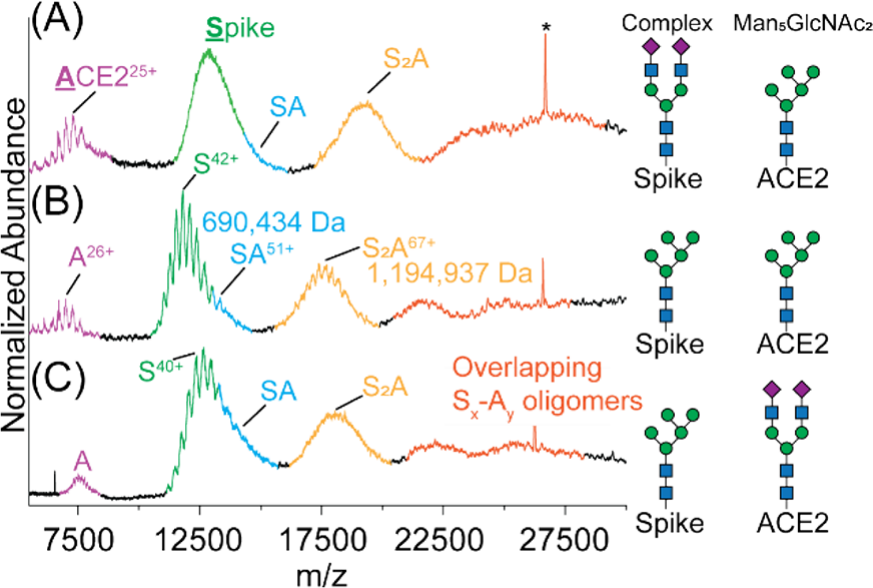
Role of *N*-glycan processing in the formation of Spike-ACE2 complexes. Native mass spectra for Spike (2 μM) and ACE2 (2 μM) with different *N*-glycan profiles: (A) complex/hybrid-type Spike and Man_5_GlcNAc_2_ on ACE2; (B) Man_5_GlcNAc_2_ on Spike and Man_5_GlcNAc_2_ on ACE2; (C) Man_5_GlcNAc_2_ on Spike and complex/hybrid-type on ACE2. Asterisk denotes a constant noise peak. Unresolved peaks are assigned to (Spike)_*x*_–(ACE2)_*y*_ complexes with high stoichiometry.

With a detailed understanding of the effects of glycosylation on isolated Spike and ACE2 complexes, we sought to evaluate the extent that glycosylation differences influence Spike and ACE2 interactions directly. We used all three possible combinations of wild-type and glycoengineered Spike and ACE2 proteins. In all three cases, after incubating equimolar concentrations of Spike with ACE2, new charge state distributions are observed consistent with the formation of larger complexes (Figure 3A–C)**.** Considering first complexes formed between Spike with native *N*-glycans and ACE2 harboring Man_5_GlcNAc_2_, the charge state series of the higher complexes were unresolved (Figure 3A). Therefore, we cannot rule out contributions from Spike dimers decorated with native glycans. By contrast, the complexes formed between Spike and ACE2, both harboring only Man_5_GlcNAc_2_ glycans, led to resolved charge states in native mass spectra (Figure 3B). Along with ACE2 dimer and Spike trimer charge states, a low abundance distribution was observed that partially overlaps with the Spike trimer (up to *m*/*z* ≈ 14,000) with a mass of 690,434 Da. A further, more prominent series of peaks, centered at *m*/*z* ≈ 17,500, corresponds to a mass of 1,194,937 Da. The former is consistent with a pentameric complex comprising a Spike trimer and an ACE2 dimer (Δ*m* ≈ 0.5%). The composition of the ∼1.2 MDa protein complex is assigned to two Spike trimers bound to dimeric ACE2. Interestingly, we did not observe any evidence for Spike dimers, ACE2 monomers or multiples of dimeric ACE2 bound to Spike, indicating that, if present, their contributions are relatively minor. Turning to other regions of the spectrum, broad features at higher *m*/*z* values of >17,500 are apparent, indicative of high-order Spike-ACE2 oligomers. Turning to the third scenario with Spike decorated by only Man_5_GlcNAc_2_ and native N-glycosylation on ACE2, despite the lack of charge state resolution, the same peak distributions persist (Figure 3C). Our observations are therefore consistent with complexes proposed from cryo-EM studies on Spike-Fab assemblies^56^ in which ACE2 can accommodate binding of two Spike trimers without steric clashes, a proposal later clarified by direct observation by cryo-EM.^16^

### Multiple Spike Trimers Bind to ACE2 Dimers

**Figure 4:**
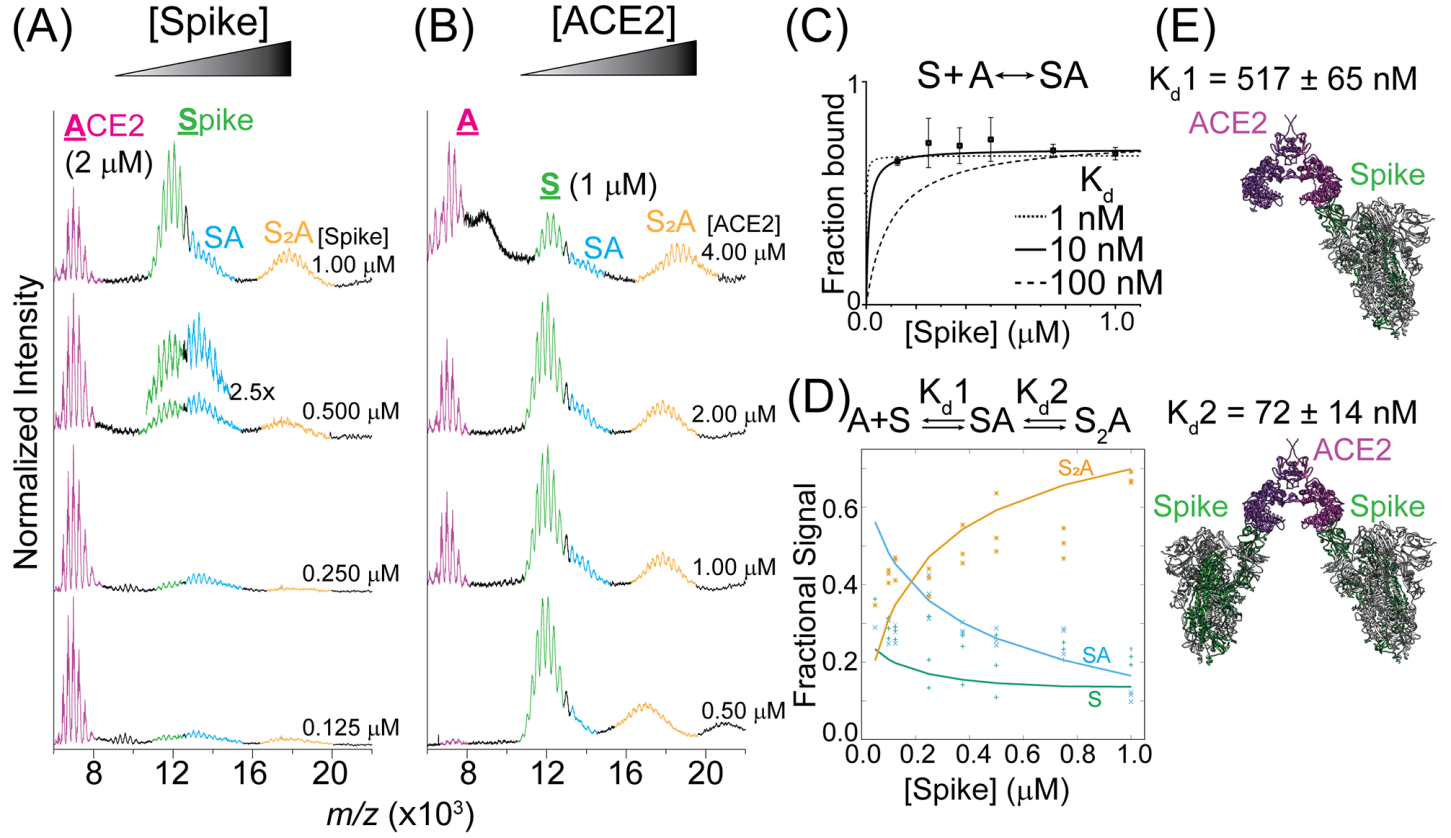
Evaluating the complexes formed between Spike and ACE2. (A) Representative native mass spectra collected at 0.125, 0.250, 0.5, and 1.0 μM Spike (bottom to top). The ACE2 concentration was held constant at 2 μM. (B) Representative native mass spectra collected at 0.50, 1.00, 2.00, and 4.0 μM ACE2 (bottom to top). The Spike concentration was held constant at 1 μM. (C) A plot of the total amount of Spike-ACE2 complex relative to free Spike captures the linear region of the binding isotherm, allowing for an estimation of the dissociation constant (*K*_d_ ≈ 10 nM). (D) Plot of the fractional signal of each complex as a function of free Spike reveals the microscopic dissociation constants for the formation of Spike-ACE2 (SA): *K*_d_1 of 517 ± 65 nM and Spike_2_-ACE2 (S2A): *K*_d_2 of 72 ± 14 nM. The probability distribution fitting leads to values for *K*_d_1 and *K*_d_2 and is used to estimate their uncertainties (Figure S3). (E) Cryo-EM models of the different ACE2-Spike organizations (PDB 7DWX).

As we had the ability to capture all forms of Spike and ACE2 as a function of their concentration in solution, we evaluated dissociation constants by recording native mass spectra at varying solution concentrations of both GNTI^–/–^ Spike and ACE2 (Figure 4A). At 1 μM Spike, the Spike_2_-ACE2 complex (*m*/*z* ≈ 17,000) was essentially equivalent in intensity to the Spike-ACE2 peak intensities (*m*/*z* ≈ 13,000). Free Spike and ACE2 were also clearly identifiable in the spectrum. As the concentration of Spike was reduced (0.500 μM) while maintaining the concentration of ACE2 constant (2.00 μM), peak intensities for Spike_2_-ACE2 decreased, whereas the Spike-ACE2 peaks showed a concomitant increase. Further lowering of the Spike concentration led to a reduction in the intensity of all complexes.

Interestingly, all higher order complexes could be observed even at the lowest Spike concentration investigated (125 nM). To consider the effect of the ACE2 concentration, we systematically explored four ACE2 concentrations (4.00, 2.00, 1.00, and 0.50 μM) while maintaining the concentration of Spike at 1.00 μM (Figure 4B). At 4.00 μM ACE2, the population of higher oligomers was the most apparent although all complexes were still detected at the lower ACE2 concentrations.

Next, we sought to characterize the dissociation constants for different Spike-ACE2 complexes by quantifying the extent of oligomer assembly as a function of Spike concentration. First, we considered the *K*_d_ for the formation of all forms of Spike-ACE2, as several literature reports have estimated this value to be sub-50 nM.^3,57,58^ By capturing the linear portion of the binding isotherm, we estimate a dissociation constant of *K*_d_ ≈ 10 nM (Figure 4C). This value is in good agreement with those previously determined (13–30 nM)^3,57,58^ indicating that even for large Spike-ACE2 complexes, relative intensities of the different species are well captured by native MS. We then expanded our calculation to examine the microscopic *K*_d_s corresponding to the formation of Spike-ACE2 and Spike_2_-ACE2. By fitting the abundance plot in Figure 4D to a binding model that encompasses each of the coupled equilibria for the formation of Spike-ACE2 and Spike_2_-ACE2, we extracted individual *K*_d_ values for the formation of these two complexes (Figure 4D). Surprisingly, the formation of Spike-ACE2 is relatively weak, *K*_d_1 = 516 ± 64 nM. By contrast, Spike_2_-ACE2 is more favorable (*K*_d_2 = 72 ± 14 nM) and is strikingly similar to the global *K*_d_—the value that represents the strength of forming all different Spike-ACE2 assemblies that we, and others, have measured. Thus, the bridging of two Spike trimers across an sACE2 dimer appears to be a favorable assembly that contributes to the overall *K*_d_ measurements reported previously. We can only speculate on the step-by-step mechanism leading to the formation of higher-order Spike-ACE2 complexes. However, the dramatic increase in interaction strength between monovalent and bivalent interactions of Spike across ACE2 (i.e., *K*_d_1 ≫ *K*_d_2), a signature of positive cooperativity, is highly suggestive that bridging of Spike trimers across sACE2 dimers may constitute one method for sACE2 to ameliorate the infectivity of the virus and potentially serves as a means for enhancing the attachment of viral-bound Spike to cACE2. We inspected the cryo-EM models to generate structural insights to understand the differences between *K*_d_1 and *K*_d_2. Each Spike trimer adopting the prefusion conformation will have at least one RBD in the up conformation, allowing it to interact with a nearby ACE2. In other words, after the formation of the initial Spike-ACE2 complex, the open binding site on the other protomer of the ACE2 dimer can be readily sequestered by an additional Spike trimer without steric hindrance.

### Role of Key N-Glycosylation Sites in Regulating ACE2 Interactions

Having demonstrated that ACE2 dimers bind multiple trimers with high affinity, we next sought to explore the impact of occupancy at specific glycosites on regulating ACE2 dimerization. We used the site-specific occupancies (Figure S2) to assign probable glycan locations in each of the ACE2 monomers we observed. Asn53, Asn90, Asn103, Asn322, and Asn564 are >95% occupied; these sites are therefore present in all forms of ACE2. Dimers of ACE2 with the lowest molecular masses will have these five glycosites occupied on each protomer. The abundance of ACE2 monomers with all seven *N*-glycans present is ∼30% relative to other ACE2 protomers. Since all N-linked sites must be occupied in this case, and given that the site-specific occupancy of Asn690 is estimated to be ca. 30% by glycoproteomics, it is clear that this entire set of glycopeptides is derived from the heaviest ACE2 glycoform. Thus, the site-specific occupancies of the remaining monomer glycoform, in which six N-linked sites are occupied, is deduced as Asn53, Asn90, Asn103, Asn322, Asn564, and Asn432. We can therefore conclude that dimerization is affected by glycan occupancy and that favorable *K*_d_s are associated with the occupancy at Asn432 and Asn690, with the former clearly playing positive role in potentiating dimerization.

**Figure 5:**
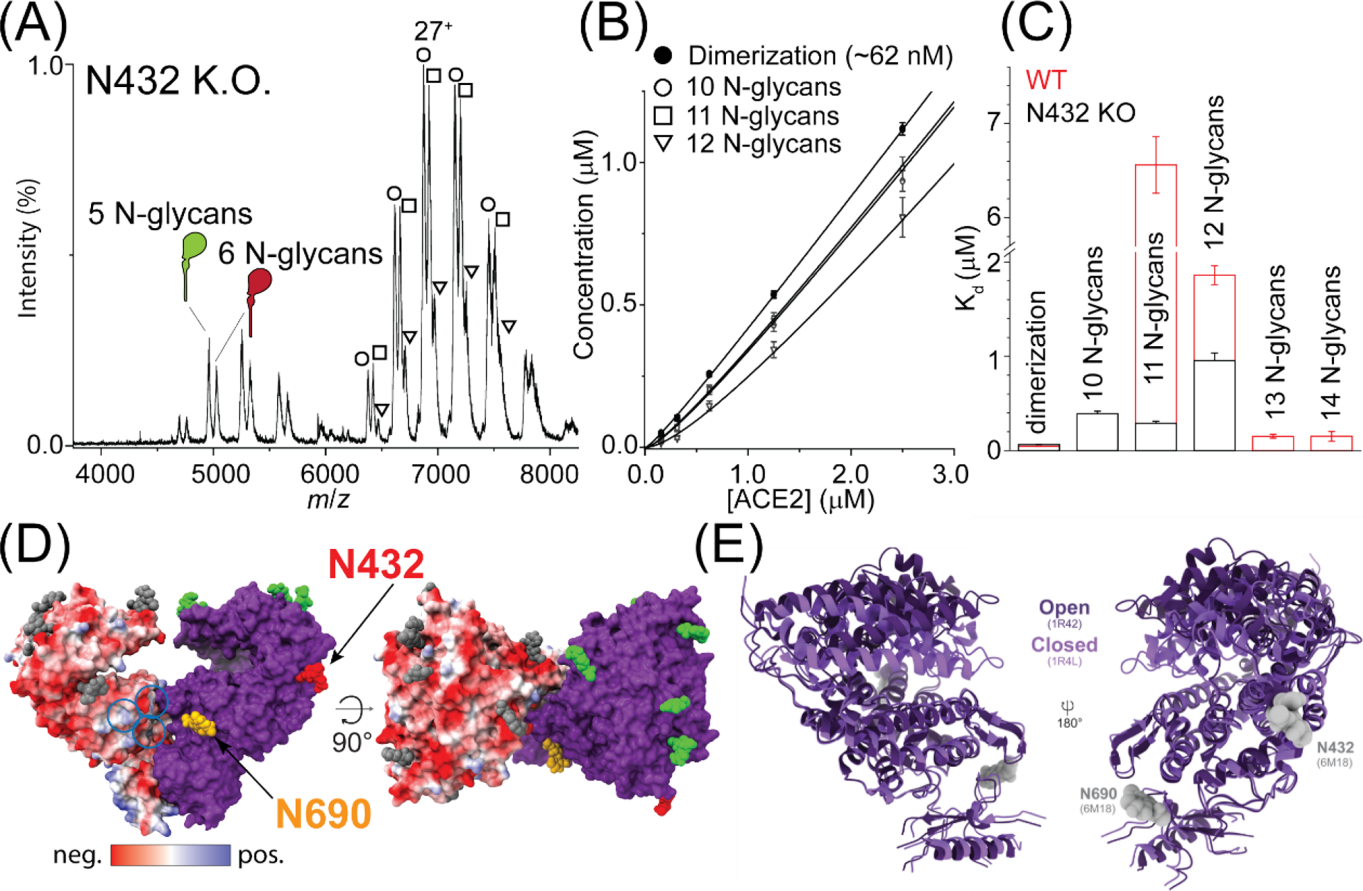
Role of Asn432 in ACE2 dimerization. (A) Native mass spectrum of ACE2 with the *N*-glycan sequon at Asn432 (N432) disrupted. Peaks denoting charge states for the different monomer and dimer glycoforms are labeled with cartoons and symbols, respectively. (B) Plot of the concentration of the different dimer glycoforms as a function of total ACE2 concentration. Lines show the best fit to a monomer–dimer equilibrium binding model. (C) Plot of each *K*_d_ for dimerization for the different ACE2 glycoforms, in the absence of Asn432 (black bars) compared to the wild-type protein (red bars). Error bars label the uncertainty for three independent measurements. (D) Overlay of the electrostatic potential onto the cryoEM map (PDB 6M18) highlighting regions of positive and negative charge (blue and red, respectively) surrounding Asn432 and Asn692. (E) Overlay of X-ray structures of ACE2 in the open (PDB 1R4L) and closed (PDB 1R42) conformations, with the glycan positions from (PDB 6M18) overlaid.

As our findings uncovered that ACE2 dimerization is sensitive to the glycosylation state of monomers, and that exceptionally favorable *K*_d_s were observed when ACE2 was glycosylated at Asn432 (6 *N*-glycans) and both Asn432 and Asn690 (7 *N*-glycans), we sought to further understand the role of these *N*-glycans in dimer formation. We generated two ACE2 constructs having Thr434 and Ser692 mutated to Ala to disrupt Asn432 (N432) and Asn690 (N690) *N*-glycan sequons, respectively. The N690 glycan knockout did not express to sufficient levels to carry out native mass spectrometry, suggesting that this site, while low in occupancy (Figure 2 and Figure S2), plays an important role in folding and trafficking. Therefore, we could not evaluate the extent of dimerization in the absence of this glycosylation site. The native mass spectrum of the Thr434Ala (N432 KO) is populated with two charge state envelopes corresponding to monomers and dimers (Figure 5A). The monomer charge state distribution for N432 KO has two peak series’ that correspond to occupancy with five and six N-glycosylation sites. As a result, the three charge state envelopes within the dimer distribution between 6500 and 8000 Th (Figure 5A), corresponding to dimers with 10, 11, and 12 *N*-glycans. The relative abundances of the monomer and dimer peaks were sensitive to total protein concentration (Figure 5B and Figure S4), which enabled us to determine the individual *K*_d_s for dimerization in the absence of glycosylation at Asn432 (Figure 5C). The formation of dimers with 10 N-glycosites (at Asn53, Asn90, Asn103, Asn322, and Asn564 in each protomer) is favorable, *K*_d_ = 390 ± 29 nM, as is the formation of dimers with 11 occupied *N*-glycans *K*_d_ = 288 ± 23 nM. As a glycan at Asn432 is absent in both cases, the only possible distribution of sugars is Asn53, Asn90, Asn103, Asn322, Asn564, and Asn690 on one protomer with the other absent of glycosylation at Asn690. Surprisingly, the addition of another *N*-glycan at Asn690 making dimers with 12 *N*-glycans has *K*_d_ = 953 ± 81 nM, further demonstrating that glycan occupancy at N690 does not potentiate dimerization.

To gain further insight into the role of each glycosite in the dimerization process, we compared the *K*_d_s between N432 KO and the WT ACE2 (Figure 5C). All the N432 KO *K*_d_s are unfavorable when compared to those of WT ACE2. Glycosylation at Asn432 is clearly important for binding. Wild-type proteins with 11 *N*-glycans (i.e., one protomer has an occupied Asn432) have an unfavorable *K*_d_, but the addition of two *N*-glycans, one at Asn432 and the other at Asn690, enhances (*viz.* decreases) the *K*_d_ by >100 fold. When the sequon at Asn432 is disrupted, the trend of decreasing (*viz.* more favorable) *K*_d_ upon additional *N*-glycans is never realized. This phenomenon is intriguing, prompting us to examine the positions of these glycans in ACE2 cryoEM^14^ and X-ray^59^ structures to further understand their roles in dimerization (Figure 5D,E). The glycosylation site at Asn690 rests along the ACE2 neck, which comprises a large portion of the dimer interface. Patches of positively charged residues across protomers can accommodate acidic sugars, leading to favorable electrostatic interactions that strengthen subunit interactions. Asn432 is distal to the dimer interface, so it is unlikely to be directly involved with scaffolding hydrogen bonding networks across protomers. Asn432 is, however, within the hinge that mediates the transition between the opened and closed states.

Importantly, only dimeric ACE2 adopting the closed conformation has been reported to bind to Spike. Many of the residues that surround the protease domain move by as much as 13 Å between the two conformations, pitching the upper lobe of the protease domain ∼16° closer to the dimer interface in the closed conformation.^59^ However, the residues of which the hinge is comprised, including Asn432, remain nearly static between the two conformations. We modeled a Man_5_GlcNAc_2_ glycan at Asn432 in these static structures and found no indications for steric clashes with proximal atoms, indicating that the glycan is capable of maintaining flexibility in both states. Indeed, MD simulations of glycosylated ACE2 found the glycan at Asn432 is highly dynamic.^20^ Thus, the glycan at Asn432 can serve as a means to remodel electrostatic interactions that differentially stabilize the dynamics between the opened–closed conformations of dimeric ACE2, similar to the “glycan gate” in Spike.^18^

**Figure 6:**
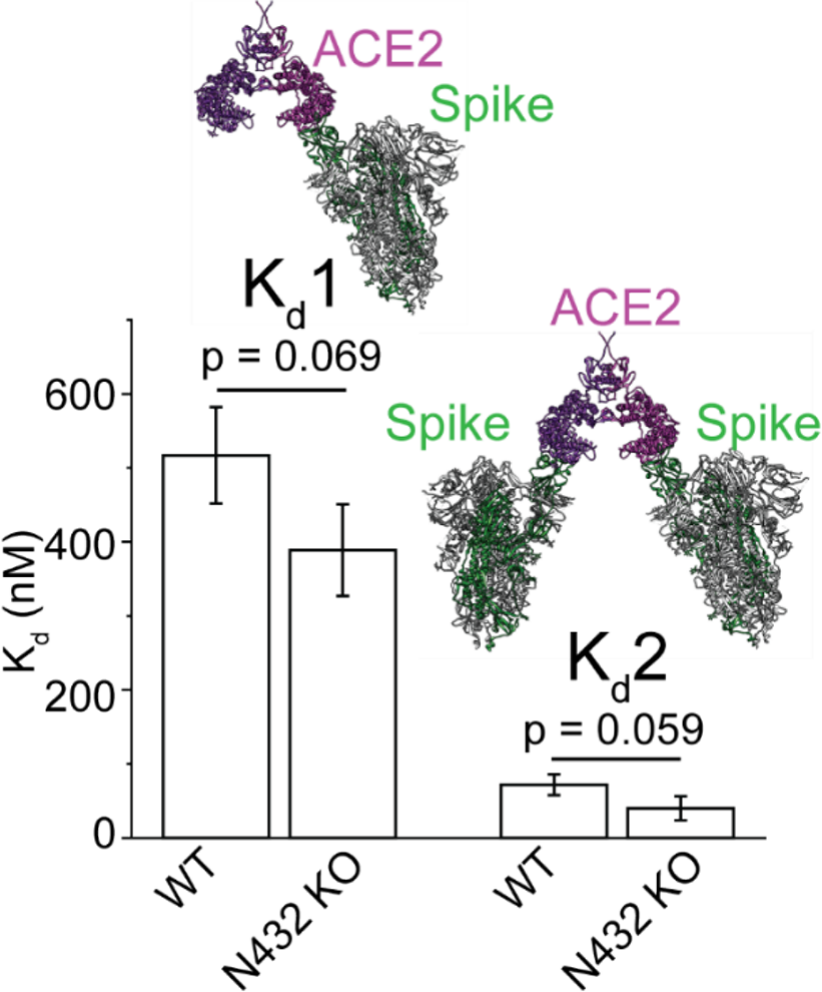
Impact of the glycan at Asn432 in the binding of Spike to ACE2 dimers. Bar plots comparing *K*_d_1 and *K*_d_2 for Spike-ACE2 and Spike_2_-ACE2 complexes, respectively, in the presence and absence of Asn432. The concentration of ACE2 was held constant (2 μM) during the assay, ensuring that dimers were the favored oligomeric state. For Spike-ACE2^WT^ complexes: *K*_d_1 = 517 ± 65 nM and *K*_d_2 = 72 ± 14 nM; Spike-ACE2^N432 KO^ complexes: *K*_d_1 = 389 ± 62 nM and *K*_d_2 = 40 ± 16 nM.

This prompted us to explore which state is stabilized in the absence of a glycan at Asn432. To probe the possibility that dimers of ACE2 N432 KO were primarily adopting the closed state, we determined *K*_d_1 and *K*_d_2 in a Spike binding assay (Figure 6). The assay conditions were identical to those involving WT ACE2 (Figure 4), so any differences in the dissociation constants can be interpreted as resulting from potential conformational differences in the ACE2 receptor. Indeed, we observed a slightly more favorable (albeit just below statistical significance threshold) *K*_d_1 and *K*_d_2 in the Spike binding assays between dimeric N432 KO relative to WT ACE2. Thus, it is likely that, in the absence of the *N*-glycan at Asn432, the closed form that favors binding to Spike is more readily adopted.

## Conclusions

In this study, we used native MS and glycoengineering to evaluate the predominant occupancy of N-linked glycans that decorate trimeric Spike proteins and ACE2. Our data is consistent with complete occupancy of Spike, which is supported by site-specific glycoproteomic analysis reported here and in previous studies.^33−38^ By employing GNTI^–/–^ cells to homogenize *N*-glycans, we have produced glycoprotein complexes, in the absence of microheterogeneity, that have allowed a straightforward correlation between glycan occupancies (from glycoproteomics), relative abundance of each glycoform (from native MS), and glycoprotein-glycoprotein interactions for macromolecular assemblies with a molecular weight of >500 kDa. The glycoengineering pipeline overcomes a large barrier that has hindered widespread native MS analyses of glycoproteins in human cell lines. While our pipeline enables the determination of interaction strengths for glycoproteins decorated with high-mannose *N*-glycans expressed in human cells, barriers for studying the interactions of endogenous glycoproteins remain.^60^ To address this limitation, we envision that the adaptation of genetic editing strategies to knockout enzymes responsible for complex *N*-glycan processing in human cell lines will allow for scalable production of glycoproteins harboring homogenous profiles of complex *N*-glycans.^61^ The combination of such genetic strategies with native MS will certainly provide a conduit for producing glycoproteins with complex glycans that are amenable to detailed biophysical study, at single glycan resolution.

Finally, our ability to distinguish the complexes formed between Spike trimers and ACE2 dimers and our evidence for Spike-ACE2 and Spike_2_-ACE2 enable us to dissect the global *K*_d_ for the microscopic binding events. It is remarkable that the former complex has a weaker dissociation constant than the latter; multivalent receptor-receptor complexes are controversial, but the evidence for cooperative formation of Spike-ACE2 complexes makes it tempting to speculate that they play an important role in receptor clustering across cellular ACE2 on the host and virus membranes. More importantly, the cooperative formation of Spike_2_-ACE2 provides experimental evidence for the potent ability of circulating sACE2 to neutralize Spikes on SARS-CoV-2. As a therapeutic, use of sACE2 has received much attention but remains controversial.^8−10,12,13^ Our results with 2.00 μM ACE2 (equivalent to 170 μg/mL, i.e., within the range required to inhibit SARS-CoV-2 infection in lung- and kidney-derived organoids (20–200 μg/mL)^8−10^ and substoichiometric Spike concentrations, showed that multiple RBDs can be sequestered. We propose that the formation of larger oligomers, which are themselves dictated by the glycan occupancy of dimeric sACE2, may constitute one mechanism for achieving neutralization of Spike by sACE2.

## Experimental Summary

Detailed experimental procedures are provided in the Supporting Information. In brief, Hexapro Spike (a kind gift from Jason McLellan, Addgene 154,754) or the ACE2 ectodomains were expressed in HEK293 GNTI^–/–^ and HEK293T cells grown as monolayers and transfected as described.^58^ ACE2 N432 KO was expressed using 293Fectin (Invitrogen) in suspension-adapted HEK293 GNTI^–/–^ cells maintained in FreeStyle media (Invitrogen). Native MS studies were carried out using an Orbitrap Q-Exactive UHMR instrument optimized for native protein analysis. LC–MS glycoproteomics was carried out using an Ultimate 3000 coupled to an Orbitrap Eclipse mass spectrometry instrument as described previously.^33,62^ Glycoproteomic spectra were searched using Byonic.

## Supporting Information Available

Detailed experimental procedures (cell culture, transfection, and protein purification conditions, instrument parameters, data acquisition parameters, and data processing); pie charts depicting glycan occupancy calculations from glycoproteomic experiments; table of intact masses of ACE2 monomers; scatter plot summarizing bootstrap analyses to estimate uncertainties; and native mass spectra showing concentration screen of ACE2 N432 KO (PDF)
