# Supplementary material for: Uncovering the Role of N-Glycan Occupancy on the Cooperative Assembly of Spike and Angiotensin Converting Enzyme 2 Complexes: Insights from Glycoengineering and Native Mass Spectrometry
Source: J Am Chem Soc. 2023 Mar 31;145(14):8021–32. doi: 10.1021/jacs.3c00291 (PMC10103161; doi:10.1021/jacs.3c00291)
Supplement: Supplementary file 1 — ja3c00291_si_001.pdf [file ja3c00291_si_001.pdf]

Supporting Information for:

Uncovering the role of N-glycan occupancy on the cooperative assembly of Spike and Angiotensin converting enzyme 2 complexes – insights from glycoengineering and native mass spectrometry

Tarick J. El-Baba,[†,‡] Corinne A. Lutomski,[†,‡] Sean A. Burnap,[†,‡] Jani R. Bolla,[†,‡,┴] Lindsay A. Baker, [‡,┬] Andrew J. Baldwin, [†,‡] Weston B. Struwe,[†,‡] Carol V. Robinson[†,‡,*]

† Physical and Theoretical Chemistry Laboratory, Department of Chemistry, University of Oxford, South Parks Road, OX1 3TA, U.K.

‡ The Kavli Institute for Nanoscience Discovery, Dorothy Crowfoot Hodgkin Building, South Parks Road, OX1 3QU, U.K.

┬ Department of Biochemistry, University of Oxford, Oxford, OX1 3QU, U.K.

┴ Present address: Department of Biology, University of Oxford, South Parks Road OX1 3RB, U.K.

*Correspondence to: carol.robinson@chem.ox.ac.uk

Experimental Procedures

Cell Culture, Protein Expression, and Purification

SARS-CoV-2 Hexapro Spike was obtained from Addgene (courtesy of Prof. Jason McLellan, Addgene 154754). The plasmid encoding residues 17 – 726 of the ACE2 ectodomain with a C-terminal hexahistadine tag was cloned into phLSEC (a kind gift from Prof. Nicole Zitzmann). To knock out N-glycosylation at Asn432, a gene encoding the ACE2 ectodomain with T434A mutation was obtained from IDT and subcloned into the phLSEC backbone. Cell lines HEK293T and HEK293S GNTI$^{-/-}$ cells were obtained from ATCC (CRL-3216 and CRL-3022, respectively). Cells were maintained in DMEM:F12 supplemented with 1X GlutaMAX, 10% fetal bovine serum, and 1x non-essential amino acids (Invitrogen). Fresh aliquots of cells were obtained for this study and were therefore not tested for the presence of mycoplasma. The evening prior to transfection, 8 – 12 x 10^6 cells were plated in a T175 flask and grown overnight. The following day, the adherent cells were washed once with ~5 mL of PBS, and then transfected with PEImax (polyscience) using a 1:3 ratio of DNA:PEI. The media was brought to ~30 mL and protein expression was allowed to commence for 48-96 hrs. Supernatants from 10 – 30 T175 flasks were pooled, clarified by centrifugation at 12,000 x g (4 °C, 30 min), passed through a 0.22 µM filter, and then either stored at -80 °C or used immediately. The ACE2 Asn432 knock out plasmid was transiently transfected into suspension adapted HEK293S GNTI-/- cells using 293Fectin (Life Technologies, Thermo Fisher Scientific) following the manufacturers' recommended protocol. On the day of transfection, ~2 x 10^6 cells/mL in 200 mL FreeStyle media (Life Technologies) was transfected as described, and protein expression was allowed to commence for 96 hrs. Supernatants were supplemented with imidazole to 20 mM before isolating the overexpressed proteins by Ni-NTA chromatography. The eluted proteins were concentrated to ~1 mg/mL using a 100 kDa MWCO (Spike) or 50 kDa MWCO (ACE2) centrifugal filter and further purified by size exclusion chromatography using a Superdex 200 Increase 10/300 column equilibrated with a buffer comprised of 20 mM Tris (pH 8.0) and 150 mM NaCl. The concentrations of each protein were determined using A_{280} measurements and calculated extinction coefficients determined by the Expasy Protparam suite (https://web.expasy.org/protparam/). Proteins were concentrated to ~10 µM, snap frozen in LN_2, and stored at -80 °C until use.

Mass Spectrometry

Native mass spectrometry experiments were carried out using an Orbitrap Q-Exactive UHMR instrument as described.[1] Prior to analysis, proteins were buffer exchanged into 350 mM ammonium acetate (pH 7.4) using Micro Zeba Spin columns with a 40 kDa MWCO (Pierce). 1-3 uL of the analyte solution was loaded into gold-coated electrospray capillaries prepared in-house for native mass spectrometry analysis. Typical electrospray parameters were: ~1.2 kV ESI voltage, 80 - 150 °C capillary temperature, 0.5 – 1 mbar backing pressure. Low-energy collisions within the in-source trapping region (20 – 50 V) and HCD cell were used to assist with thermalizing the high molecular weight ions: in-

source trapping voltage: 20 – 50 V, HCD energy: 20 V. Data were obtained in the positive ion mode at a resolving power of 25,000 (at m/z 200). For Spike-ACE2 binding experiments, the buffer-exchanged proteins were combined in the desired ratios and incubated for ~10 min at room temperature before analysis. The instrument was operated in “low mass” detection mode in the ACE2 dimerization studies. All other assays were carried out using the “high mass” detection mode. For equilibrium binding assays, the solutions were mixed by gentle pipetting and allowed to sit at room temperature for ~15 min to equilibrate prior to analysis.

Glycoproteomics

To prepare glycopeptides, ~15 μg of protein was treated with 8M urea for 10 min while periodically vortexing to induce unfolding. Disulfide bonds were reduced with 20 mM TCEP at 56 °C for 45 min. The reduced disulfide bonds were alkylated with 20 mM iodoacetamide in the dark for 1 hr. The urea was diluted to 1 M and sequencing grade trypsin (Promega) was added at a 1:20 w/w ratio. Tryptic peptides were generated by incubating the solutions overnight at 37 °C. Tryptic peptides were desalted using C18 stage tips (Pierce) and analyzed using an Orbitrap Eclipse Tribrid platform as reported previously.[2] Glycopeptide data was analyzed with Byonic and Byologic (Protein Metrics) using the search parameters described by Burnap and Struwe.[3] All glycopeptide assignments were manually validated. For quantification, the extracted ion chromatogram intensities for each glycopeptide and unoccupied peptides were summed and plotted relative to the total intensity for each glycosite. For proteins expressed from HEK293S GNTI$^{-/-}$ cells, all glycopeptides were identified as high-mannose, consistent with previous glycomics work which showed that all N-glycans are $Man_5GlcNAc_2$ for Spikes and ACE2 proteins.[3] However, we opted to not restrict the glycopeptide databases used in searching these datasets; all 37 common N-linked glycans were searched as common modifications.

Data Analysis

Data were plotted in OriginPro. Native mass spectra in Fig 1 were modelled essentially as described[4] using a resolution parameter that corresponded to a peak width of 180 Th. The ACE2 spectrum in Fig 2A was deconvoluted using UniDec with default parameters except for the following that were empirically optimized: smoothing of 2; no basline subtraction; mass range of 6000 – 8000; charge range of 5 – 50; Mass Range 5000 – 500,000; Sample mass every 10 Da; width 1 Th. The mass distribution was then plotted in OriginPro.

To generate the bar plots in Fig 2B, the relative quantity of each dimer glycoform resulting from self-association of each monomer **G**lycoform ($G1, G2, G3$) was determined mathematically, akin to predicting isotope ratios.[5] Briefly, a polynomial expansion of the monomer glycoforms was used to identify the likelihood of observing a given dimer state.The peak areas for the three different monomeric ACE2 **G**lycoforms ($G1, G2, G3$)

were determined and then normalized to determine their probabilities (p_1, p_2, p_3), where $p_1 + p_2 + p_3 = 1$.

G1 is the monomer glycoform with the lowest mass and *G3* the highest mass. The six distinct dimer masses that result from pairwise combinations of *G1, G2, and G3* were determined.

For example,

$$G1 + G1 = G1G1$$

To determine the probability of observing any dimer glycoform from the pairwise association of the pool of monomers, a polynomial expansion of monomer probabilities was determined:

$$(p_1 + p_2 + p_3)(p_1 + p_2 + p_3)$$

$$(p_1{}^2 + p_2{}^2 + p_3{}^2 + 2p_1p_2 + 2p_1p_3 + 2p_2p_3)$$

For example, the probability of observing *G1G1* is $p_1{}^2$. The probability distribution was plotted in Fig 2B as gray bars.

To determine the dissociation constants (K_ds) for ACE2 dimerization, the concentrations of all species were plotted against the total protein concentration and fit to an equilibrium binding model essentially as described.[6] The same procedure was followed to determine the overall (global) K_d for the formation of Spike-ACE2 complexes; the fractional signal of a respective complex was plotted against the respective protein concentration, and fit to a single site equilibrium binding model using OriginPro or a Python script. For the global Spike-ACE2 K_d, only the linear region of the binding isotherm was captured by the experimental data. An upper estimate of ~10 nM on the modelled K_d was therefore reported – nearly identical to the values reported in literature using various techniques.[7,8,9] Nevertheless, we modelled isotherms assuming K_d values of 1 nM and 100 nM to demonstrate that the measured K_d is indeed near 10 nM. This exercise demonstrated that the peak areas for the different species identified in our native mass spectrometry assays captured the accepted behavior.

To determine the K_d1 and K_d2 for the formation of Spike-ACE2 and Spike2-ACE2, respectively,

$$S + A \leftrightarrow SA \quad K_d1 = \frac{S \cdot A}{SA} \qquad (1)$$

$$SA + S \leftrightarrow S2A \quad K_d2 = \frac{SA \cdot S}{S2A} \qquad (2)$$

the fractional signal in each complex was determined from the experimental data, where *S*, *A*, *SA*, and *S2A* are the fractional signal of free Spike, free ACE2 dimers, Spike-ACE2,

and Spike$_2$-ACE2 in each native mass spectrum. K_d1 and K_d2 are the equilibrium dissociation constants for SA and $S2A$, respectively.

In all experiments, the quantity of ACE2 and Spike could be accounted for using:

$$A_{tot} = A + SA + S2A \qquad (3)$$

$$S_{tot} = S + SA + 2S2A \qquad (4)$$

A_{tot} and S_{tot} are molar concentrations of ACE2 and Spike in each experiment. Equations (1) and (2) were rearranged and substituted into (3) and (4) to generate master equations which allow for solving of SA and $S2A$ at any concentration of S_{tot} or A_{tot}. It is difficult to accurately capture changes in signal abundances for ions < 8000 Th when transmitting and detecting large *m/z* ions (>12,000 Th). Therefore, we opted to hold the concentration of ACE2 constant. This made it straightforward to account for A, the fraction of unbound ACE2, across serial dilutions of S_{tot}. In addition, we only quantified complexes that had resolved peaks corresponding to a charge state distribution that could be assigned. The master equations were encoded into Python and solved for K_d1 and K_d2 as described.[10] Fitting performed by minimizing the χ^2 value. After fitting, the goodness of fit was further validated by analyzing the uncertainties in the K_d values via bootstrap analysis with 10,000 replicates (**Fig. S3**).

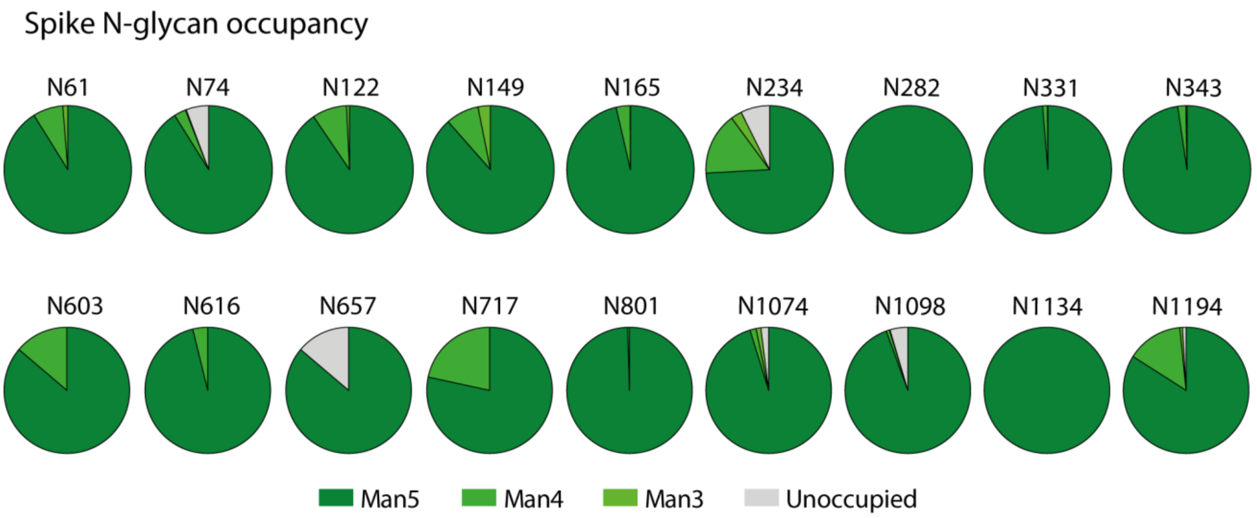

Fig. S1. N-glycan occupancy of Spike determined by glycoproteomic analysis. All N-glycans were identified as high-mannose.

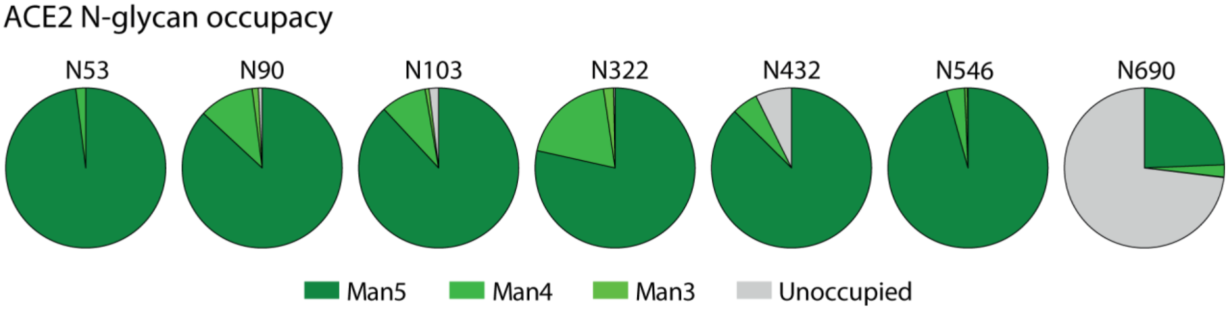

Fig S2. Site-specific N-glycan occupancies of ACE2. All glycans were identified as high-mannose.

Table S1. Masses of monomeric ACE2.

	Monomeric ACE2 Masses		
**N-glycoform**[a]	*Expected (Da)*[b]	*Measured (Da)*	*Δm (%)*[c]
**5**	89200	88794	0.46
**6**	90416	90040	0.42
**7**	91632	91366	0.29

[a] each $Man_5GlcNAc_2$ contributes 1216 Da to sequence mass
[b] expected mass assuming processing of signal peptide results in construct containing residues 18-726 and known cofactors Zn^{2+} and Cl^- were not included in the theoretical mass calculation.
[c.] The deviation is a result of not including Zn^{2+} and Cl^- adduction in the theoretical mass calculation

Table S2. Masses of monomeric ACE2 Asn432 KO.

	**Monomeric ACE2 Masses**		
**N-glycoform**[a]	*Expected (Da)*[b]	*Measured (Da)*	*Δm (%)*[c]
**5**	89274	89278	<0.01
**6**	90490	90518	0.03

[a] each $Man_5GlcNAc_2$ contributes 1216 Da to sequence mass
[b] expected mass assuming processing of signal peptide results in construct containing residues 18-726 and known cofactors Zn^{2+} and Cl^- were not included in the theoretical mass calculation.
[c.] The deviation is a result of not including Zn^{2+} and Cl^- adduction in the theoretical mass calculation

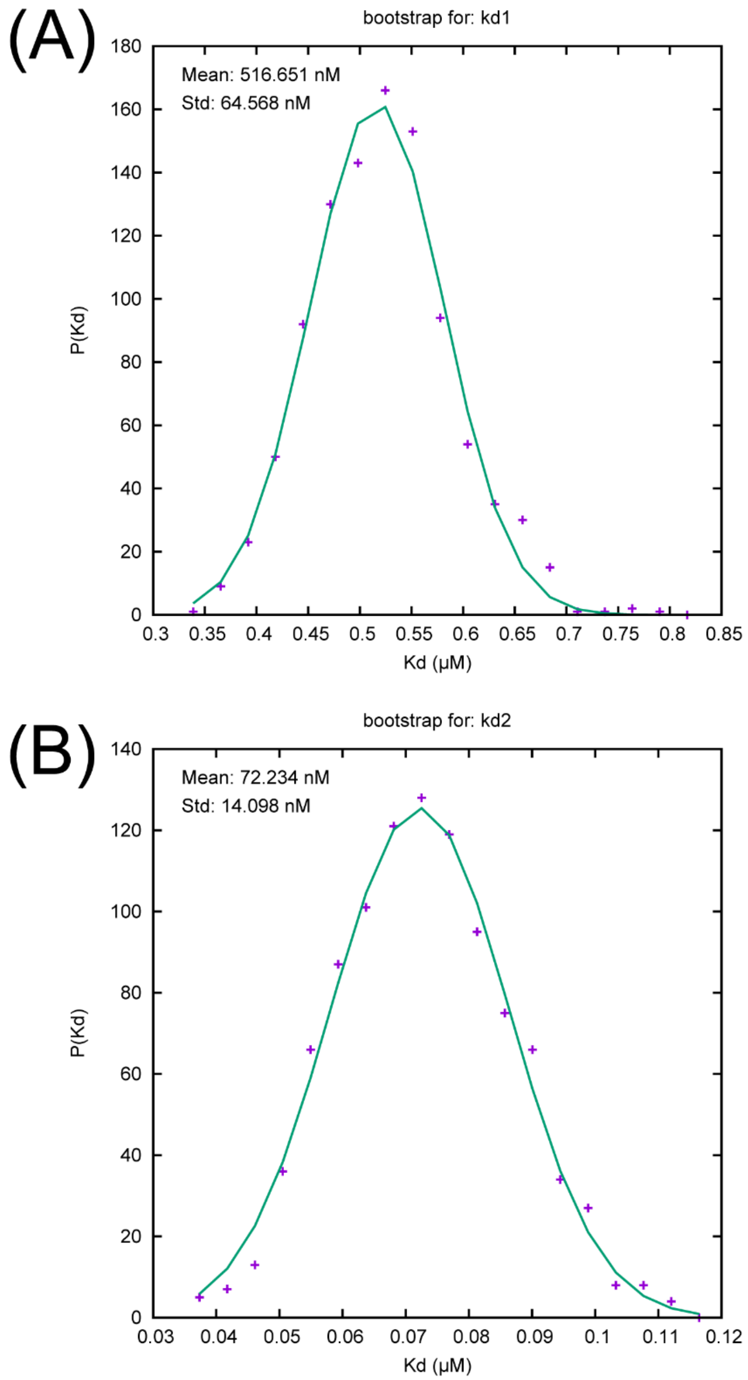

Fig S3. Analysis of the uncertainties in (A) K_d1 and (B) K_d2 by bootstrap analysis (10,000 replicates).

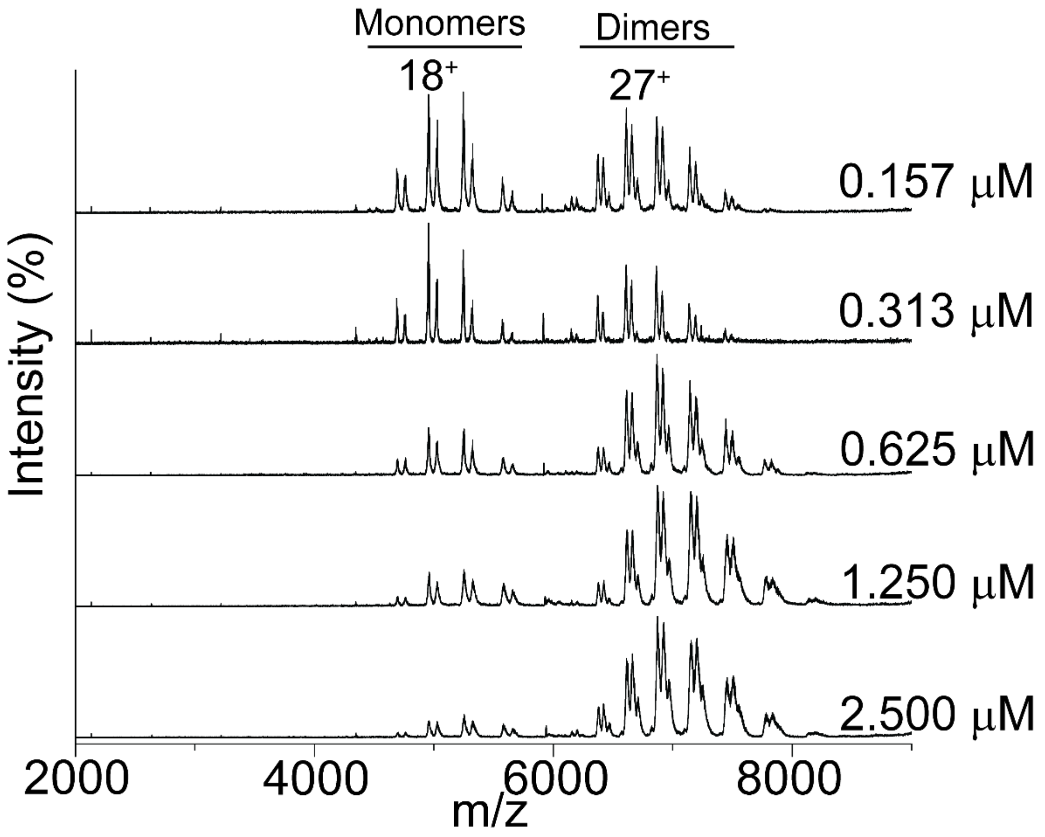

Fig S4. Native mass spectra for several representative concentrations of ACE2 N432 KO. Values denote the concentration of monomer.

Supplementary References

1 Waterbeemd, M. V. D.; Ford, K. L.; Boll, D.; Reinhardt-Szyba, M.; Routh, A.; Makarov, A.; Heck, A. J. R. Nat Methods. **2017**, *3*, 283-286.

2 Lutomski, C. A.; El-Baba, T. J.; Bolla, J. R.; Robinson, C. V. *JACS Au* **2021,** *1*, 1147-1157.

3 Burnap, S. A.; Struwe, W. B., Mass photometry reveals SARS-CoV-2 spike stabilisation to impede ACE2 binding through altered conformational dynamics. *Chem Commun (Camb)* **2022,** *58* (93), 12939-12942

4 Allison, T. M.; Reading, E.; Liko, I.; Baldwin, A. J.; Laganowsky, A.; Robinson, C. V. *Nat. Commun.* **2015**, *8551*.

5 Yergy, J. A. Int. J. Mass Spectrom. Ion Phys. **1983**, *52*, 337-349.

6 Bergdoll, L. A.; Lerch, M. T.; Patrick, J. W.; Belardo, K.; Altenbach, C.; Bisignano, P.; Laganowsky, A.; Grabe, M.; Hubbell, W. L.; Abrason, J. *Proc. Natl. Acad. Sci. USA.* **2017**, *115*, E172-E179.

7 Wrapp, D.; Wang, N.; Corbett, K. S.; Goldsmith, J. A.; Hsieh, C. L.; Abiona, O.; Graham, B. S.; McLellan, J. S., Cryo-EM structure of the 2019-nCoV spike in the prefusion conformation. *Science* **2020,** *367* (6483), 1260-1263.

8 Schaub, J. M.; Chou, C. W.; Kuo, H. C.; Javanmardi, K.; Hsieh, C. L.; Goldsmith, J.; DiVenere, A. M.; Le, K. C.; Wrapp, D.; Byrne, P. O.; Hjorth, C. K.; Johnson, N. V.; Ludes-Meyers, J.; Nguyen, A. W.; Wang, N.; Lavinder, J. J.; Ippolito, G. C.; Maynard, J. A.; McLellan, J. S.; Finkelstein, I. J. *Nat Protoc* **2021,** *16* (11), 5339-5356.

9 Sztain, T.; Ahn, S. H.; Bogetti, A. T.; Casalino, L.; Goldsmith, J. A.; Seitz, E.; McCool, R. S.; Kearns, F. L.; Acosta-Reyes, F.; Maji, S.; Mashayekhi, G.; McCammon, J. A.; Ourmazd, A.; Frank, J.; McLellan, J. S.; Chong, L. T.; Amaro, R. E. *Nat Chem* **2021,** *13* (10), 963-968.

10 Hochberg, G. K. A.; Shephard, D. A.; Marklund, E. G.; Santhanagoplan, I.; Degiacomi, M. T.; Laganowsky, A.; Allison, T. M.; Basha, E.; Marty, M. T.; Galpin, M. R.; Struwe, W. B.; Baldwin, A. J.; Vierling, E.; Benesch, J. L. P. *Science*, **2018**, 359 (6378), 930-935.
